# Analysis of Molecular Biomarkers in Resected Early-Stage Non-Small Cells Lung Cancer: A Narrative Review

**DOI:** 10.3390/cancers14081949

**Published:** 2022-04-13

**Authors:** Filippo Tommaso Gallina, Luca Bertolaccini, Daniele Forcella, Shehab Mohamed, Serena Ceddia, Enrico Melis, Francesca Fusco, Claudia Bardoni, Daniele Marinelli, Simonetta Buglioni, Paolo Visca, Federico Cappuzzo, Lorenzo Spaggiari, Francesco Facciolo

**Affiliations:** 1 Thoracic Surgery Department, IRCCS Regina Elena National Cancer Institute, 00144 Rome, Italy; daniele.forcella@ifo.it (D.F.); enrico.melis@ifo.it (E.M.); francesco.facciolo@ifo.it (F.F.); 2 Department of Thoracic Surgery, IEO, European Institute of Oncology IRCCS, 20100 Milan, Italy; luca.bertolaccini@gmail.com (L.B.); mohamed.shehab@gmail.com (S.M.); claudia.bardoni@ieo.it (C.B.); lorenzo.spaggiari@ieo.it (L.S.); 3 Medical Oncology 2, IRCCS Regina Elena National Cancer Institute, 00144 Rome, Italy; serena.ceddia@uniroma1.it (S.C.); francescafusco322@gmail.com (F.F.); federico.cappuzzo@ifo.it (F.C.); 4 Medical Oncology Unit B, Policlinico Umberto I, Sapienza University, 00185 Rome, Italy; daniele.marinelli@uniroma1.it; 5 Department of Pathology, IRCCS Regina Elena National Cancer Institute, 00144 Rome, Italy; simonetta.buglioni@ifo.it (S.B.); paolo.visca@ifo.it (P.V.)

**Keywords:** NSCLC, early stage, EGFR, NGS, KRAS, ALK, PD-L1, tumour mutational burden

## Abstract

**Simple Summary:**

In the last few years, the treatment of advanced NSCLC has radically changed after the development of new drugs against specific molecular targets. Moreover, multiple tumour biopsies have become mandatory in order to better select the appropriate targeted therapy. Molecular analysis using NGS in the early stage of NSCLC is still relatively widespread. The recent clinical trials that use targeted therapies in neoadjuvant and adjuvant settings also require molecular characterisation for early-stage patients. Due to the widespread use of molecular analysis in patients with early-stage NSCLC, the prognostic role of molecular biomarkers needs to be fully understood. This paper aimed to review the most recent studies associating the molecular expressions of early-stage NSCLC with survival.

**Abstract:**

Next-generation sequencing has become a cornerstone in clinical oncology practice and is recommended for the appropriate use of tailored therapies in NSCLC. While NGS has already been standardised in advanced-stage NSCLC, its use is still uncommon in the early stages. The recent approval of Osimertinib for resected EGFR-mutated NSCLC in an adjuvant setting has launched the hypothesis that other targeted therapies used in metastatic patients can also lead to improved early-stage outcomes of NSCLC. The impact of molecular biomarkers on the prognosis of patients undergoing radical surgery for NSCLC is still unclear. Notably, the heterogeneous populations included in the studies that analysed surgical patients could be the main reason for these results. In this review, we report the most important studies that analysed the impact of principal molecular biomarkers on the survival outcomes of patients who underwent radical surgery for NSCLC.

## 1. Introduction

Worldwide, non-small cell lung cancer (NSCLC) remains the primary cause of cancer mortality [1]. While significant improvement has been achieved over the last decade, the median survival of patients with metastatic illness remains dismal, with just a tiny percentage of patients living more than two years [2]. Routine molecular pathology testing for lung cancer began in earnest in 2004, after it was discovered that patients who responded dramatically to the EGFR tyrosine kinase inhibitors (TKIs), gefitinib and erlotinib, also had detectable EGFR-activating/sensitising mutations in their tumour biopsy specimens [3]. Since then, new medications (for example, crizotinib for ALK-positive lung cancer) have been developed in reverse by first investigating disease biology to identify genetic lesions in lung cancer, and then designing a therapy to target those lesions [4]. This bidirectional method of identifying and verifying novel therapeutic targets has resulted in rapid progress for patients with stage IV lung cancer, the efficacy of which can be confirmed within weeks of commencing treatment. Along with EGFR, ALK, and PD-1/PD-L1 signalling pathway inhibition, medicines targeting ROS1, RET, BRAF V600E, and HER2 are now considered the standard of care for tracking genetic modifications over time, and serial biopsies are now considered the standard of care for tracking genetic alterations over time. Numerous prospective clinical studies have established the benefits of molecular pathology diagnoses and targeted treatment selection in stage IV lung cancer [5].

This review aims to examine the available evidence regarding the prognostic and predictive utility of biomarker mutations in early-stage non-small cell lung cancer.

## 2. Materials and Methods

**Figure 1:**
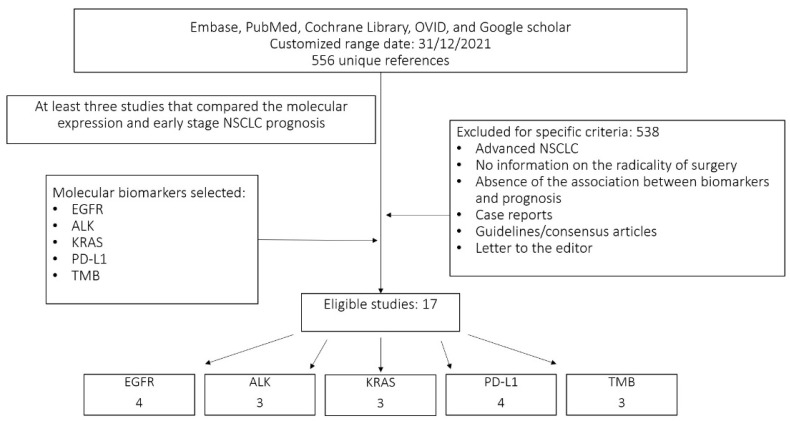
Flow diagram for the evaluated studies.

We performed a bibliographic search of studies that analysed the principal molecular biomarkers investigated in early-stage NSCLC. We analysed only biomarkers present in at least three studies that associated the prognosis of early-stage NSCLC with molecular expression. The biomarkers analysed were: EGFR, ALK, KRAS, PD-L1 and TMB. To begin, we conducted a comprehensive search of Embase, PubMed, the Cochrane Library, OVID, and Google Scholar up to December 2021, using a combination of keywords and related terms for ‘non-small cell lung cancer’, ‘epidermal growth factor receptor’, ‘k-ras’, ‘k-ras mutations’, ‘erlotinib’, ‘Osimertinib’, ‘gefitinib’, ‘cetuximab’, ‘anaplastic lymphoma kinase’, ‘alectinib’, ‘programmed cell death-ligand 1’, ‘nivolumab’, ‘pembrolizumab’ and ‘tumour mutational burden’. The search was restricted to articles written in the English language. The electronic search was supplemented by a manual search of meeting abstracts (World Conference on Lung Cancer, European Society of Medical Oncology Annual Congress, American Society of Clinical Oncology Annual Meeting, and European Lung Cancer Conference) and reference lists for original and review articles. Then, all selected papers were grouped in an Excel file to remove the duplicates, and the studies that did not compare the molecular expression and the DFS or OS of early-stage NSCLC were consequently excluded. The remaining studies were analysed for associated information. The flow chart of the extracting article is reported in Figure 1.

Inclusion criteria for this study were:

(1) At least three studies associating the prognosis of early-stage NSCLC with specific molecular expression;
(2) The study was a randomised control trial, prospective study, retrospective study or review article;
(3) Presence of molecular analysis using NGS or PCR methods;
(4) Patients underwent radical surgery for early-stage NSCLC;
(5) An association between molecular expression and overall or disease-free survival.

The exclusion criteria were:

(1) No information regarding the radicality of the surgery;
(2) Advanced NSCLC;
(3) Absence of a specific association between biomarkers and prognosis;
(4) Case reports;
(5) Guidelines/consensus articles;
(6) Letters to editors.

## 3. Results

### 3.1. Epidermal Growth Factor Receptor (EGFR)

EGFR is a major oncogenic driver in NSCLC and can often be muted in lung adenocarcinoma [6]. The prevalence of EGFR mutations in lung adenocarcinoma (LUAD) can considerably change concerning the population analysed. Indeed, when compared to the western population, EGFR mutant LUADs are considerably more frequent in the East Asian population [7]. The analysis of the subsequent studies confirmed that, in Asian countries, EGFR mutations were found in about half of patients with LUAD, while in western countries, the percentage of EGFR mutations was around 10%; accordingly, the rates of non-smokers among EGFR-positive NSCLC patients were significantly higher than in patients with EGFR wild-type tumours [8].

Exon 19 deletions and the exon 21 L858R mutation were the most frequently observed EGFR changes, both predictive of susceptibility to anti-EGFR tyrosine kinase drugs (TKIs). Around 10% of EGFR-mutated tumours contain rare somatic mutations, which are more or less evenly distributed among the affected exons (18, 19, 20, and 21) [9]. Compared to specific EGFR mutations, evidence on the treatment sensitivity of NSCLCs harbouring rare EGFR mutations was restricted to a few case reports and a few small clinical studies. In some circumstances, almost all uncommon EGFR mutations responded to standard EGFR inhibitors, while they were often less susceptible to TKIs than ex19del and L858R mutants [10].

The therapeutic landscape after complete tumour resection in oncogene-addicted early-stage NSCLC has been significantly reshaped in the last few years. Osimertinib was the first EGFR TKI to be licensed for the adjuvant treatment of patients with EGFR-mutated NSCLC following resection based on the results of the ADAURA trial [11]. Patients in stage Ib-IIIa treated with Osimertinib showed lower rates of recurrence when compared with placebo; however, mature data on overall survival from this trial are eagerly awaited, given the negative results from previous trials of anti-EGFR TKIs in resected NSCLC [12]. Even though EGFR mutations proved to be both predictive and prognostic in advanced NSCLC, previous studies in early-stage NSCLC reported controversy regarding the prognosis according to EGFR status [13].

Zhang et al. performed one of the first systematic reviews and meta-analyses to detect the prognostic value of EGFR mutations in patients with resected NSCLC. They selected studies from different countries and confirmed the differences in terms of prevalence between continents. The analyses of DFS and OS did not show any differences according to EGFR status [14]. The studies are resumed in Table 1.

Yang et al., in a non-interventional study, analysed the EGFR mutation status and survival outcomes in patients with LUAD stage I to III, completely resected and operated, across 26 sites in China. A total of 472 patients were finally included in the study, and most of them underwent lobectomy (91.5%). Regarding the EGFR status, 260 patients showed an EGFR mutation, with the most common being Ex19 deletions and the Ex21 L858R mutation. Although in the first analysis of the ICAN study, the authors found that, in EGFR mutant patients, the recurrence rate was higher than in the wild type; in the extended analysis, long-term outcomes did not show any differences between the two groups in terms of DFS and OS across the postoperative stages. Interesting results were shown in terms of adjuvant chemotherapy. Indeed, there were no survival differences between groups according to EGFR status in patients who underwent adjuvant therapy compared to patients who were not treated. The survival analysis did not show any differences between the subgroups of EGFR mutant patients or wild type in the group of patients who did not receive adjuvant chemotherapy [15].

Chen et al. conducted a retrospective study to determine the prognostic value of EGFR mutations in patients with pT1a and pT1b invasive lung adenocarcinoma. EGFR mutation was detected in 216 patients and was significantly more frequent in never smokers, females, lepidic pattern, acinar pattern, and papillary predominant pattern adenocarcinoma. In this analysis, no differences were reported in recurrence between patients with and without EGFR mutations. However, EGFR mutant patients showed prolonged overall survival in the entire cohort and in the analysis of the subgroups [16].

The analysis of NGS performed by Jao et al., regarding 214 specimens from patients across stages I–III, reported EGFR mutations in 12% of samples. Any mutation was also associated with significantly worse DFS after adjusting for smoking status, stage, and histology. However, no differences in terms of OS were found. Besides, no patient analysed in this study had been subjected to TKI therapy [17].

A large cohort of patients with EGFR mutations analysed the recurrence rate compared to EGFR wild-type patients. Additionally, in this study, the prognostic role of EGFR alteration remained controversial. Indeed, resected EGFR-positive NSCLC was associated with a comparable, if not more significant, risk of recurrence compared to wild-type EGFR-positive NSCLC. However, this did not transfer into a worse OS [18].

Although several studies have investigated the prognostic role of EGFR mutations in surgically resected patients, the results are still controversial. Indeed, some studies support the hypothesis that EGFR mutations could be associated with favourable outcomes, while others report either no significant association or association with an unfavourable prognosis. The heterogeneous populations analysed could represent a reason for the different results among these studies. Therefore, while EGFR alterations are significantly higher in the Asian population compared to the Caucasian population, the reasons for racial differences in EGFR mutation frequency remain unknown.

The heterogeneous surgical patients included in the studies could be another reason why the outcomes are unclear. Indeed, little information about the preoperative staging, the kind of surgery, the lymphadenectomy, and the surgery’s radicality has been reported. Given the paradigm shift introduced by the approval of Osimertinib in the postoperative setting, an accurate depiction of the prognostic impact of EGFR mutations in NSCLC is critical.

### 3.2. Anaplastic Lymphoma Kinase (ALK)

Rearrangements of the anaplastic lymphoma kinase (ALK) gene are well-established risk factors for non-small cell lung cancer (NSCLC) [19]. The sequential use of ALK-directed tyrosine kinase inhibitors (TKIs) has been shown to significantly enhance outcomes in patients with metastatic ALK-positive NSCLC. It is considered to be the gold standard of therapy [20]. The therapeutic algorithm of the metastatic setting is well defined; nevertheless, the rarity of ALK fusions in non-metastatic NSCLC represents a challenge for the development of clinical trials in the early-stage setting.

In previous reports, ALK rearrangements in early-stage NSCLC were associated with a trend towards poorer outcomes compared with other clinically relevant genomic subsets (e.g., EGFR, KRAS mutation).

In a recent paper, the authors identified the genomic alterations contributing to disease recurrence and analysed early-stage lung adenocarcinoma with a low tumour burden. In the multivariate analysis, ALK fusions, ROS Proto-Oncogene 1 (ROS1), and proto-oncogene RET were recognised as independent prognostic genetic factors of recurrence [21].

Dou et al. described immunohistochemistry (IHC)-confirmed early-stage lung cancer with an intergenic-breakpoint ALK fusion based on previous studies which described worse postoperative outcomes in patients with ALK rearrangement early-stage NSCLC; adequate treatment should be provided to reduce postoperative recurrence [22].

Considering early-stage NSCLC, ALK fusion has demonstrated a worse recurrence-free survival (RFS) than EGFR mutation, despite differences in OS not being reported. In 2018, Chaft et al. found 764 patients with stage I–III NSCLC who had had a surgical resection. They observed that the median RFS was 24.3 months in patients with ALK rearrangement, but this was not attained in patients with EGFR mutations, and was 72.9 months in patients with KRAS mutations [23].

A retrospective study including 309 patients with resected stage IA lung adenocarcinoma showed that ALK fusion was associated with a higher risk of disease and more frequent recurrence in regional lymph nodes than ALK-negative tumours [24].

Therefore, ALK rearrangements may indicate the patient’s prognosis even in the early stages and guide postoperative treatment (Table 2).

ALCHEMIST (NCT02194738) and ALINA (NCT03456076) are two phase III clinical trials currently enrolling patients in the adjuvant setting for ALK rearrangement. ALK+ patients were randomised to receive crizotinib versus observation in the first study of alectinib, or gold-standard treatment in the ALINA study, with up to 24 months of treatment in the absence of disease progression or unacceptable toxicities. The trials’ primary objectives are overall survival (OS) and disease-free survival (DFS) [25,26].

Accordingly, more clinical trials are needed with the aim of better understanding early-stage ALK-positive NSCLC. A comprehensive molecular analysis of the entire population of resected lung tumours is necessary—new generation platforms based on the multigene analyses of DNA and RNA should be exploited to highlight the presence of ALK fusions in early-stage tumours [27].

### 3.3. Kirsten Rat Sarcoma (K-RAS)

Table 3 summarises the studies that were analysed. A prospective cohort of 365 patients treated at Massachusetts General Hospital for resected early-stage NSCLC evaluated the effect of K-RAS mutations on survival [28]. K-RAS mutations were detected in 22.1% of patients with adenocarcinoma histology, and the analysis was confined to this subgroup. Women had a higher prevalence of mutations than men (26.2% versus 17.4%), and mutations were discovered exclusively in smokers. Survival was related to K-RAS mutation (*p* = 0.009, log-rank test). Only stage I was found to be significantly related to a worse result (*p* = 0.002, log-rank test). Multivariate analysis indicated that the K-RAS mutation, with a risk ratio of 1.8, remained a statistically significant independent predictor of bad outcomes. These findings contrast a previous report that evaluated the prognostic significance of a panel of six biomarkers in completely resected stage I NSCLC, including: death-associated protein kinase promoter methylation; interleukin-10 protein expression; cyclooxygenase-2 messenger RNA expression; human telomerase reverse transcriptase catalytic subunit mRNA expression; retinoic acid receptor-beta mRNA expression; and K- patients. These were tracked for at least five years; K-RAS mutations were discovered in 34% of samples, but did not affect disease-specific survival (*p* = 0.998) or overall survival (*p* = 0.517). Without targeted therapy, 3-year OS rates were 90%, 76%, and 66% for patients with an EGFR mutation, EGFR/K-RAS wild type, and K-RAS mutant, respectively. Although the multivariate analysis corroborated this tendency, it was not statistically significant [29].

A more realistic strategy is to employ genetic tests to detect common genetic variants, such as KRAS and EGFR mutations, and integrate histologic and mutational data to differentiate lung tumours. Combining histologic and mutational data to classify lung tumours has demonstrated that the classic Martini–Melamed criteria are insufficient in up to 30% of instances. A 2014 study published in the Annals of Surgical Oncology examined 131 patients with multiple primary lung tumours and discovered that histologic mutational approaches had a predictive value superior to the Martini–Melamed criteria [30]. However, despite the widespread availability of KRAS and/or EGFR mutation testing, these genetic abnormalities have been discovered in a subset of resected lung adenocarcinomas and not in squamous carcinomas. In ordinary practice, the multidisciplinary team, led by the attending pathologist, determines when and whether genetic studies are necessary to differentiate numerous primary malignancies from the metastatic disease on an individual basis [31].

According to a meta-analysis of systematic reviews, K-RAS mutations are associated with poor prognosis in non-small cell lung cancer patients, particularly those with adenocarcinoma and early-stage NSCLC. Thus, detecting K-RAS mutations may aid us in stratifying individuals at high risk and developing targeted treatment for them [32].

### 3.4. Programmed Cell Death-Ligand 1 (PD-L1)

The analysis of PD-L1 expression on tumour cells has gained particular interest [33]. Indeed, the blockade of the programmed cell death 1 (PD-1)/PD-1 ligand 1 (PD-L1) signalling pathway through monoclonal antibodies is the most common approach to improve immune response against several types of cancers [34]. Over the last few years, the monoclonal antibodies that act on this pathway have become increasingly common in advanced NSCLC treatment. It is also widely used in adjuvant and neoadjuvant treatments in patients with totally resected NSCLC with a high expression of PD-L1 [35,36]. The relationship between PD-L1 expression and the prognosis of radically resected early-stage NSCLC patients could increase the efficacy of these drugs before or after surgery, and could allow us to better select patients that would benefit from these treatments [37].

Recently, the predictive significance of PD-L1 expression in NSCLC patients has been investigated, notably in the advanced stages. Additionally, prior research has established that PD-L1 predicts poor prognosis and survival in these individuals [38]. However, the use of PD-L1 expression in predicting prognosis in patients with early-stage NSCLC who have received complete resection remains debatable. The studies that were analysed are summarised in Table 4.

Cooper et al. published one of the first studies examining the prevalence of PD-L1 expression in early-stage NSCLC in 2015. They looked at relationships between clinicopathologic characteristics and patient outcomes [39]. PD-L1 expression was estimated to be 50%. In 32.8% of patients, PD-L1 expression of any intensity was detected. Moreover, 7.4% of NSCLCs expressed a high level of PD-L1. A higher level of PD-L1 expression was associated with younger patient age and a higher grade of tumour (*p* ≤ 0.05). In the multivariate analysis, patients with high PD-L1 expression showed significantly longer overall survival (*p* ≤ 0.05).

Shi et al. have published a meta-analysis examining the effect of PD-L1 expression on the prognosis of resected NSCLC at an early stage. We analysed 15 trials with a total of 3790 patients [40]. The pooled HR demonstrated that PD-L1 expression was associated with a considerably shorter DFS (HR = 1.56, 95% CI: 1.18–2.05, *p* = 0.01) and a significantly worse OS (HR = 1.68, 95% CI: 1.29–2.18, *p* = 0.01). The correlations between PD-L1 expression and TNM stage (I vs. II–III: OR = 0.45, 95% CI:0.34–0.60, *p* = 0.000), smoking status (Yes vs. No: OR = 1.43, 95% CI:1.14–1.80, *p* = 0.002), and lymph node metastases (N+ vs. N: OR = 1.97, 95% CI:1.26–3.08, *p* = 0.003) were examined.

In 2018, D’Arcangelo et al. examined the expression of PD-L1 in 289 early-stage, surgically resected NSCLC that had not received adjuvant treatment [41]. The PD-L1 assessment was characterized in this study as follows: (1) PD-L1 high (tumour percentage score, TPS50%), PD-L1 low (TPS 1–49%), and PD-L1 negative (TPS1%); (2) PD-L1 positive (TPS50%) and negative (TPS50%); and (3) as a continuous variable. The analysis of OS and DFS concerning the PD-L1 expression did not show differences between groups. A strong association was detected between the tumour grade and the PD-L1 expression. Additionally, the analysis of patients with grade 3 tumours did not show a difference in outcome between high PD-L1 and low PD-L1.

The authors have summarised the published studies on the prognostic significance of PD-L1 expression in NSCLC. Regarding the effects of resected early-stage NSCLC, controversies remain. Indeed, although some authors reported a poor prognosis in patients with a high expression of PD-L1, others showed no significance in terms of prognosis.

Sum JM et al. sought to determine the predictive significance of PD-L1 expression in a large cohort of patients with non-small cell lung cancer (NSCLC) [42]. The codification of the PD-L1 expression cut-off, based on the clinical trial assay, was proposed to allow for the most accurate prediction of clinical response in patients with non-small cell lung cancer treated with pembrolizumab. Among the 520 patients with stage I tumours, disease recurrence was more common in the 176 patients who tested strongly or weakly positive for PD-L1 (48% versus 27% in the 344 patients who tested negative for PD-L1; *p* = 0.001). Positive PD-L1 status, whether strong or mild, was likewise related to a shorter DFS than negative PD-L1 status (*p* = 0.0001). However, the link was not statistically significant when adjusting for postoperative chemotherapy or radiotherapy.

We hypothesise that these different results may partly refer to the not-homogeneous populations analysed. The kind of surgery performed could widely impact the prognosis compared to the molecular expression, and little information was provided about the specific surgeries in most of these studies. Another factor that may represent a confounder is adjuvant therapy. As reported by Sum JM et al. after the adjustment for the postoperative adjuvant treatment, the prognostic role of the PD-L1 expression loses its value. Indeed, to understand the role of the PD-L1 expression on prognosis, other studies should be conducted using a more homogenous population of surgically resected patients.

#### Tumour Mutational Burden (TMB)

Tumour mutational burden (TMB) represents the total number of non-synonymous mutations of a tumour genome. In the last few years, several clinical studies have shown a significant correlation between TMB and the outcomes of patients who underwent immunotherapy for multiple tumour types. TMB seems to be an effective biomarker that could also predict immunotherapy’s efficacy for NSCLC. Meanwhile, for patients with radically resected NSCLC, TMB can help to evaluate long-term prognosis [43].

One of the first studies to examine the role of TMB was published in 2015. Indeed, Rizvi et al. revealed that a greater frequency of non-synonymous mutations was linked with an enhanced objective response, durable clinical benefit (DCB), and progression-free survival (PFS) in patients with non-small cell lung cancer (NSCLC) who underwent anti-PD-1 therapy. The authors speculated that the link between high TMB levels and pembrolizumab efficacy could be explained by the detection of neoantigens formed due to somatic mutations, which could impede anti-PD-1 therapy activity [44].

The survival benefit of adjuvant chemotherapy is still poor, and further studies should be performed to determine which kind of patients can fully benefit from adjuvant chemotherapy. Moreover, the analysis of TMB in a patient with radically resected NSCLC can detect new markers of prognosis [45].

Devarakonda et al. recently used a targeted panel of 1538 genes to analyse 908 specimens from patients who underwent surgery for NSCLC. Treatment, age, sex, performance score, histology, type of surgery, and stage were all corrected. The results indicated that patients with resected NSCLC who had a high non-synonymous TMB had a better survival prognosis. The advantage of adjuvant treatment was more evident in patients with a low TMB [46].

Tian et al. performed a study to construct a TMB estimation model based on genomic data from the TGCA cohort and validated it using three independent cohorts. This analysis showed that the DFS was higher in the estimated TMB high-group, and OS was comparable in the two groups in the early-stage cohort. The analysis of the advanced stage confirmed that TMB could serve as a predictive biomarker for anti-PD-1 and anti-PD-L1 treatment responses [47].

In 2018, Owada-Ozaki et al. published one of the first studies calculating TMB within individual tumours using whole-exome sequencing and next-generation sequencing in patients with early-stage NSCLC who had had surgery. A total of 90 patients were examined, and the results showed that high TMB could be associated with a poor prognosis (HR = 6.633, *p* = 0.0003). Despite the total cohort of patients, no differences were found in DFS. Analysing only the stage I NSCLC, the recurrence rate was significantly higher in the high-TMB group [48]. These findings placed the TMB evaluation as a critical step after the surgical resection of NSCLC. Namely, given the high-TMB as a predictor of the efficacy of immune checkpoint inhibitors, this therapy used in an adjuvant setting could improve recurrence rate and survival (Table 5).

## 4. Conclusions

Molecular pathology testing is not yet acceptable for all patients with resectable stages of illness (stage I–III). There is rising evidence that genetic testing can help differentiate several primary cancers amenable to surgical excision. The EGFR mutation is quite frequent (10%), has significant prognostic implications, and presents an opportunity to enrol all EGFR-mutant patients in clinical trials or evaluate novel therapies in high-risk patients. Although ALK and ROS1 gene rearrangements are uncommon, they signal a possibility for clinical trial enrolment or novel treatment in high-risk individuals. The immune checkpoint inhibitors targeting PD-1/PD-L1, after the relevant results in the treatment of advanced NSCLC, have also been tested in several clinical trials in adjuvant or neoadjuvant settings. The results are encouraging, but other studies should be performed to better select the patients that could benefit from this treatment.

In the last few years, together with biomarker analysis performed on tissue samples, circulating tumour DNA has been investigated to better understand the molecular behaviour of NSCLC [49]. The early assessment of this relevant marker can predict recurrence in early-stage and locally advanced NSCLC, which could lead to the prompt start of specific targeted therapies [50]. Regarding the prognostic role of the ctDNA, the results in the literature are still unclear. However, some evidence suggests that perioperative ctDNA could be associated with oncological outcomes [51].

The papers we reported in this review showed different outcomes regarding the prognostic roles of the most common molecular biomarkers. Other studies should be conducted to better evaluate this critical benchmark. Indeed, to evaluate the prognostic role of a molecular biomarker in a surgical population, the inclusion criteria of the patients should be stringent, avoiding patients that have undergone suboptimal surgical treatment. The focus of drug development in early-stage illness has moved away from existing treatment and towards the principles of cancer biology and tumour molecular categorisation. In the future, crucial molecular testing for lung cancer will be prognostic. However, it will also be utilised to determine which medications should be employed in multidisciplinary treatment and perioperative systemic therapy selection.

## Figures and Tables

**Table 1:** Disease-free survival (DFS) and overall survival (OS) in early-stage NSCLC with EGFR mutations.

Study	Year	Kind of Study	Patients (Total/EGFR)	Stage	DFS	OS
Zhang et al.	2014	Meta-analysis	3337 (n.r.)	I–III	n.s. ^1^	n.s.
Yang et al.	2021	Retrospective	476 (260)	I–III	n.s.	n.s.
Chen et al.	2021	Retrospective	338 (216)	I	n.s.	positive
Jao et al.	2018	Retrospective	214 (46)	I–III	Negative	n.s.

^1^ n.s.: not significant. n.r.: not reported.

**Table 2:** Disease-free survival (DFS) and overall Survival (OS) in early-stage NSCLC with ALK expression.

Study	Year	Kind of Study	Patients (Total/ALK)	Stage	DFS	OS
Kim et al.	2021	Retrospective	203 (12)	I–II	negative	n.s. ^1^
Chaft et al.	2018	Retrospective	764 (29)	I–III	negative	n.s.
Shin et al.	2018	Retrospective	309 (23)	I	negative	n.s.

^1^ n.s.: not significant.

**Table 3:** Disease-free survival (DFS) and overall survival (OS) in early-stage NSCLC with KRAS expression.

Study	Year	Kind of Study	Patients (Total/K-Ras)	Stage	DFS	OS
Nelson et al.	1999	Prospective	365 (44)	I–IV	n.r.*	negative
Lu et al.	2004	Retrospective	94 (34)	I	n.s.	n.s.
Lin M-W et al.	2014	Retrospective	64 (4)	I (syn)	n.s.	n.s.

* n.r.: not reported. n.s.: not significant.

**Table 4:** Disease-free survival (DFS) and overall survival (OS) in early-stage NSCLC with PDL-1 expression.

Study	Year	Kind of Study	Patients	Cut-Off	Stage	DFS	OS
Cooper et al.	2015	Retrospective	678	≥50%	I–III	n.r. ^1^	positive
Shi et al.	2021	Retrospective	3790	variable	I–III	negative	negative
D’Arcangelo et al.	2019	Retrospective	289	≥50%	I–III	n.s.	n.s.
Sum JM et al.	2016	Retrospective	520	1–95%/≥96%	I–III	negative	n.s.

^1^ n.r.: not reported. n.s.: not significant.

**Table 5:** Disease-free survival (DFS) and overall survival (OS) in early-stage NSCLC with high TMB.

Study	Year	Kind of Study	Nr of Patients	DFS	OS
Devarakonda et al.	2018	Prospective	908	n.r.*	positive
Tian et al.	2020	Retrospective	1026	positive	n.s.
Owada-Ozaki et al.	2018	Retrospective	90	n.s.	negative

* n.r.: not reported. n.s.: not significant.
